# Procedures to evaluate potential of plants as natural food preservatives: Phytochemical characterization, novel extraction technology, and safety evaluation—A comprehensive review

**DOI:** 10.1002/fsn3.4303

**Published:** 2024-07-04

**Authors:** Negin Ahmadi, Nazanin Mosleh, Mahta Yeganeh, Nadia Ahmadi, Sara Malakouti, Saleh Shahsavari, Reza Shahraki, Somaye Katebi, Mina Agapoor, Sonia Sadeghi, Karim Bagheri

**Affiliations:** ^1^ Department of Food Science and Technology Islamic Azad University, Science and Research Branch Tehran Iran; ^2^ Department of Food Science and Technology, Faculty of Agriculture University of Tabriz Tabriz Iran; ^3^ School of Medicine Shahid Beheshti University of Medical Sciences Tehran Iran; ^4^ General Bureau of Standard Sistan and Baluchestan Province Iran National Standards Organization Zahedan Iran; ^5^ Department of Horticultural Science, Faculty of Agriculture Urmia University Urmia Iran

**Keywords:** advanced extraction technologies, *Artemisia*, medicinal herbs, natural antimicrobials, natural antioxidants

## Abstract

There is increasing demand for natural food preservative in food manufacturing industry as it is the key to meet consumers' preferences toward healthier food choice. Plant is listed among the most important resources of bioactive components to be utilized as the green and natural food preservatives. There are more than 10,000 kinds of bioactive components in plants that possess antioxidant and antimicrobial properties. *Artemisia* with potential antimicrobial and antioxidant attributes, as well as functional and medicinal properties, is one of the most important plant species. The manuscript presents a comprehensive review of the potential of the *Artemisia* species as natural food preservatives. The current challenges and ways forward in using *Artemisia* EOs and extracts as food preservatives are also discussed. This topic is timely and important considering the natural preservatives used to replace chemical ingredients, sustaining quality, healthy properties, and shelf life of food products as well as efficient and novel extraction techniques.

## Graphical abstract

The use of plant extracts and EOs as preservatives in food products is a growing trend. *Artemisia* revealed good antimicrobial and antioxidant effects. *Artemisia* can be a novel natural agent for food preservation. The recent developments in extraction of *Artemisia* are discussed.

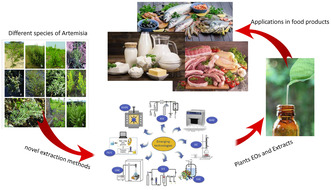

## 1 INTRODUCTION

The dependence of people being on plant kingdom for different purposes such as fodder, food, medicine, fuel, etc., is as old as the presence of humans in this universe (Koul & Taak, 2018). Natural ingredients due to their few side effects, and as a result less toxic, are preferred mostly by consumers compared to chemically synthesized ingredients (Haile & Jiru, 2022). Therefore, medicinal herbs can potentially be utilized as a source of healthy products for the production and improvement of functional foods. *Asteraceae* family species, with high antioxidant and antimicrobial properties, are listed among the most practical and useful plants to produce a healthy diet (Kazeminia et al., 2022). *Asteraceae* includes many species with economic and medicinal significance, and is known as the largest eudicot angiosperm family with ~500 species. *Artemisia* is the leading genus of *Asteraceae* family (Bora & Sharma, 2011). The genus *Artemisia*, which comprises hardy herbs and shrubs mostly covered with hairs, belongs to the tribe *Anthemideae* of the family *Asteraceae* (Compositae). Due to the medicinal value as well as the commercial and economic significance of *Artemisia*, their proper chemical identification is of great importance. There are more than 600 secondary metabolites with valuable medical properties which have been identified from *Artemisia* species (Shahrivari et al., 2022). These essential metabolites may include saponins, EOs, cyanogenic glycosides, flavonoids, tannins, phenols, unsaturated lactones, glucosinolates, and phenolic glycosides (Al‐Zubairi et al., 2011). The *Artemisia* extracts have been reported to be rich in compounds like flavonoids and phenolic with medicinal properties such as antinociceptive, antimalarial, antimicrobial, antihepatotoxic, antihelmintic, antidiabetic, antifungal, antihysteric, antioxidants, diuretic, stimulant, and digestive (Mehani et al., 2018). Some species of *Artemisia* genus have been utilized not only to treat epilepsy, psychosis, depression, diabetes, insomnia, irritability, stress, and anxiety but also as flavoring in cake, pastries, tobacco, etc. (Babayan et al., 2022; Pavithra et al., 2018). *Artemisia* essential oil is not only a rich source of β‐thujone, camphor, 1,8‐cineole, and α‐thujone but also has high antioxidant and antibacterial attributes (Baldino et al., 2017). In this regard, techno‐functional attributes of *Artemisia* have been proved in foods, like Hy‐line Brown male chickens' breast (Wan et al., 2017), rainbow trout fillets (Raeisi et al., 2020), yogurt (Zedan et al., 2021), soybean oil (Ayoughi et al., 2011), and thigh muscles in broilers (Baldino et al., 2017). Some of the most notable *Artemisia* species include, but are not limited to *A. annua*, *A. argyi*, *A. vulgaris*, *A. herba‐alba*, *A. absinthium L*, *A. pallens*, *A. afra*, and *A. japonica* (Bora & Sharma, 2011).

The aim of the present work is to investigate the potential of the *Artemisia* species as a natural food preservative.

## 2 METHODOLOGY

### 2.1 Investigation of the most important *Artemisia* species

#### 2.1.1 Artemisia annua

The common name of *A. annua* is *sweet annie*, *sweet wormwood*, or *wormwood*. *A. annua* is an erect, branched, annual herb with a sweet aromatic odor having very slender stem approximately 2 m high (Zeb et al., 2019). *A. annua* has naturalized worldwide and has expanded in various areas such as Switzerland, the USA, Burma, Brazil, Thailand, Malaysia, Australia, Central and South Europe, India, South China, Afghanistan, Iran, and Pakistan. The twig and leaf extract of *A. annua* is a rich source of artemisinin, 3‐O‐β‐d‐glucopyranoside of sitosterol, chrysosplenetin, scopoletin, and eupatin (Patel, 2022). *A. annua* is widely utilized to make flavored drinks, fragrant wreaths, and EOs distillation for industrial and perfumery usage. *A. annua* has also been reported to have antiviral, anticholesterolemic, antimicrobial, anti‐inflammatory, anticonvulsant, antiplasmodial, antitumor, antimalaria, and antihyperlipidemic properties (Mirghaed et al., 2020; Wang et al., 2020; Yan et al., 2019). Furthermore, *A. annua* leaf extract has been exhibited to increase the *Oreochromis niloticus* immunity (Soares et al., 2020), enhance the *Cyprinus carpio* antioxidant capacity (Mirghaed et al., 2020), and improve the growth performance of *Oncorhynchus mykiss W* (Koshinski, 2018). *A. annua* received increased importance when the sesquiterpene lactone artemisinin, as the active principle of *A. annua* against malaria, was isolated from this species (Feng et al., 2020). *A. annua* is listed among the top 10 industrial crops around the world.

#### 2.1.2 Artemisia argyi

*A. argyi*, known as a perennial herbaceous plant of *Compositae artemisia*, is widely distributed in most areas of China. *A. argyi*, which is known as medical grass, with high resistance against drought and cold weather likes wet and warm conditions (Zhang et al., 2018). The dried leaf of *A. argyi* is widely utilized for the treatment of menstrual‐related symptoms, hemostasis, inflammation, diarrhea, and eczema in many countries (Liu et al., 2021; Shin et al., 2017). According to modern pharmacological studies, *A. argyi* due to the abundance of volatile terpenoids can be utilized as a broad‐spectrum antiviral and antibacterial treatment (Jiang et al., 2019). Furthermore, the extracted active substance from *A. argyi* leaves, due to their pleasant aroma, has wide uses as additives to cosmetics and functional foods (Anwar et al., 2016). Chemical studies indicated the presence of several secondary metabolic components such as organic acids, coumarins, sesquiterpene lactones, polysaccharides, flavonoids, and terpenoids in *A. argyi* (Han et al., 2017; Jiang et al., 2019).

#### 2.1.3 Artemisia vulgaris

*A. vulgaris*, which spreads rapidly upon introduction by a well‐developed rhizome system, is a broadleaf perennial approximately 2.5 m tall and 75 cm width (Ekiert et al., 2020). *A. vulgaris*, which develops fruit called achenes, is well established to cool weather with gravelly, sandy, and well‐drained soils (Ekiert et al., 2020). Pandey et al. (2017) also analyzed *A. vulgaris* EOs composition and listed the following major components: germacrene D (8.42%), borneol (4.44%), camphor (11.89%), chrysanthenone (4.48%), beta‐thujone (19.99%), and sabinene (11.29%) (Pandey et al., 2017). The EOs of *A. vulgaris* mainly include volatile components, such as 1,8‐cineole, β‐caryophyllene, α‐thujone, camphor, camphene, germacrene D, and α‐pinene (Judžentienė & Buzelytė, 2006). In traditional medicine, *A. vulgaris* is utilized as infusions to which stomachic, cytostatic, antibacterial, anthelmintic, antitumor, and antipyretic actions have been attributed (Blagojević et al., 2006).

#### 2.1.4 Artemisia herba‐alba

*A. herba‐alba*, also known as desert wormwood, is a greenish‐silver perennial herb that grows approximately 20–40 cm in height (Moufid & Eddouks, 2012). This plant is a chamaephyte and is distributed in most of Europe, Saudi Arabia, and North Africa (Libya). *A. herba‐alba* is widely utilized to treat stomach problems such as abdominal pain and diarrhea and heal exterior wounds (Sapkota et al., 2022). Furthermore, *A. herba‐alba* has exerted potent vaso‐relaxant, antifungal, antispasmodic, and antibacterial activities (Mohamed et al., 2010; Skiker et al., 2010). Studies on *A. herba‐alba* indicated that this plant is among the most practical utilized aromatic herbs to counter the threat of COVID‐19 (Asdadi et al., 2020; Brahmi et al., 2022; Hasan et al., 2022). *A. herba‐alba* by apoptosis induction, DNA destruction, and reducing cell viability in cancer cells has shown a potential cytotoxic activity against different cancers such as myelogenous leukemia, bladder carcinoma, laryngeal carcinoma, and human colon cancer cell (Tilaoui et al., 2015). Identification of *A. herba‐alba* EOs indicated the presence of sesquiterpenes, monoterpenes, flavonoids, phenolic components (cirsilineol and hispidoline), and cis‐chrysanthenyl acetate, as well as oxygen‐containing monoterpenes (77.3%) such as chrysanthenone, a‐thujone, and camphor (Belhattab et al., 2014).

#### 2.1.5 *Artemisia absinthium* L (wormwood)

*A. absinthium*, which is traditionally known as *Ariti*, is typically identified as a perennial and erect herb about 30–60 cm tall (Mohammed et al., 2022). This plant is distributed in North Africa, West Asia, and Europe (Szopa et al., 2020). *A. absinthium* in traditional medicine is used for treating stomach ache, worms, fevers, and sepsis, and acts as a diuretic (Koul & Taak, 2018). Mohammed et al. (2022) indicated the presence of terpenoids, phenols, proteins, quinines, tannins, carbohydrates, flavonoids, and alkaloids by the phytochemical analysis of *A. absinthium* extract. The main constituents of *A. absinthium* EOs (isolated from the aerial parts) are linalyl 3‐methylbutanoate (7.5%), sabinene (8.1%), trans‐sabinyl acetate (8.8%), cis‐â‐epoxyocimene (10.7%), and â‐thujone (19.8%) (Blagojević et al., 2006). Identification of semisaturated azulenes obtained from *A. absinthium* EOs; for instance, guaiol (0.49%), chamazulene (3.38%), γ‐gorgeonene (0.86%), and α‐gorgeonene (7.15%), an indication of the plant's responsibility in the biosynthesis of azulenes as its main sesquiterpene components (Mohammed et al., 2022). This plant has been utilized in the preparation of antimicrobial, anti‐inflammatory, anthelmintic, and anticold drugs due to its stimulant, carminative, digestive, antidepressant, antiseptic, and choleretic impacts (Goud et al., 2015). It has been indicated that terpenoid, thiophene, flavonoid, and phenolic are listed among the main secondary metabolites of this genus (Yamari et al., 2004).

#### 2.1.6 Artemisia pallens

*A. pallens* known as “Davana “is an aromatic, erect herb, approximately 60 cm tall, with small yellow flowers and much divided leaves which grows in the temperate Himalayas (Durgadevi et al., 2022). *A. pallens* is a high‐value annual fragrant herb cultivated commercially in South India (Dongare, 2022). This plant is a versatile medicinal herb utilized for treating a variety of ailments especially diabetes (Kumar & Kumud, 2010). *A. pallens* also has been utilized for the treatment of blood pressure, depression, cold, cough, and measles (Pavithra et al., 2018). This plant contains phenolics and ascorbic acid both of which are powerful antioxidants.

#### 2.1.7 Artemisia afra

*A. afra*, approximately 2 m tall, is a perennial woody shrub with ridged, hairy, and leafy stem (Van Wyk, 2008). This plant is distributed in the high land areas (1500 and 3000 m) of Southern Africa and Eastern altitudes with loamy sands, volcanic ash, or calcareous clay loams of granitic or volcanic origin. *A. afra* is a popular medicinal herb which commonly called African wormwood (Van Wyk, 2011). *A. afra* is widely distributed in South Africa from the Mountains of Cederberg in the Cape to tropical East Africa, northwards and stretching as far north as Ethiopia (Van Wyk, 2008). Evaluation of the preliminary phytochemical screening of the petroleum ether, dichloromethane, and ethanol extract of *A. afra* leaves indicated the presence of steroids, cardiac glycosides, phenolic quinones, coumarins, terpenoids, anthraquinones, saponins, tannins, flavonoids, and alkaloids, while there were no anthocyanins in all crude extracts (Yimam & Desalew, 2022). *A. afra* has long been utilized to treat different sicknesses like dyspepsia, malaria, coughs, diabetes, cold, headaches, and disorders of bladder and kidney (Patil et al., 2011). du Toit and van der Kooy (2019) indicated good to moderate activity of *A. afra* against Leishmania donovani, Trypanosoma species, parasitic gastrointestinal nematodes, and Plasmodium falciparum (du Toit & van der Kooy, 2019).

#### 2.1.8 Artemisia japonica

*A. japonica* is native to Viet Nam, Korea, China, and Japan. *A. japonica* is widely utilized to treat tuberculosis, hypertension, malaria, headache, and fever (Vo, 1997). This plant is used as a tea and vegetable in Chongqing, Fujian, and other places. It has been declared beneficial for liver disorders such as jaundice and viral hepatitis (Lu et al., 2023).

### 2.2 Advanced extraction technologies

**FIGURE 1:**
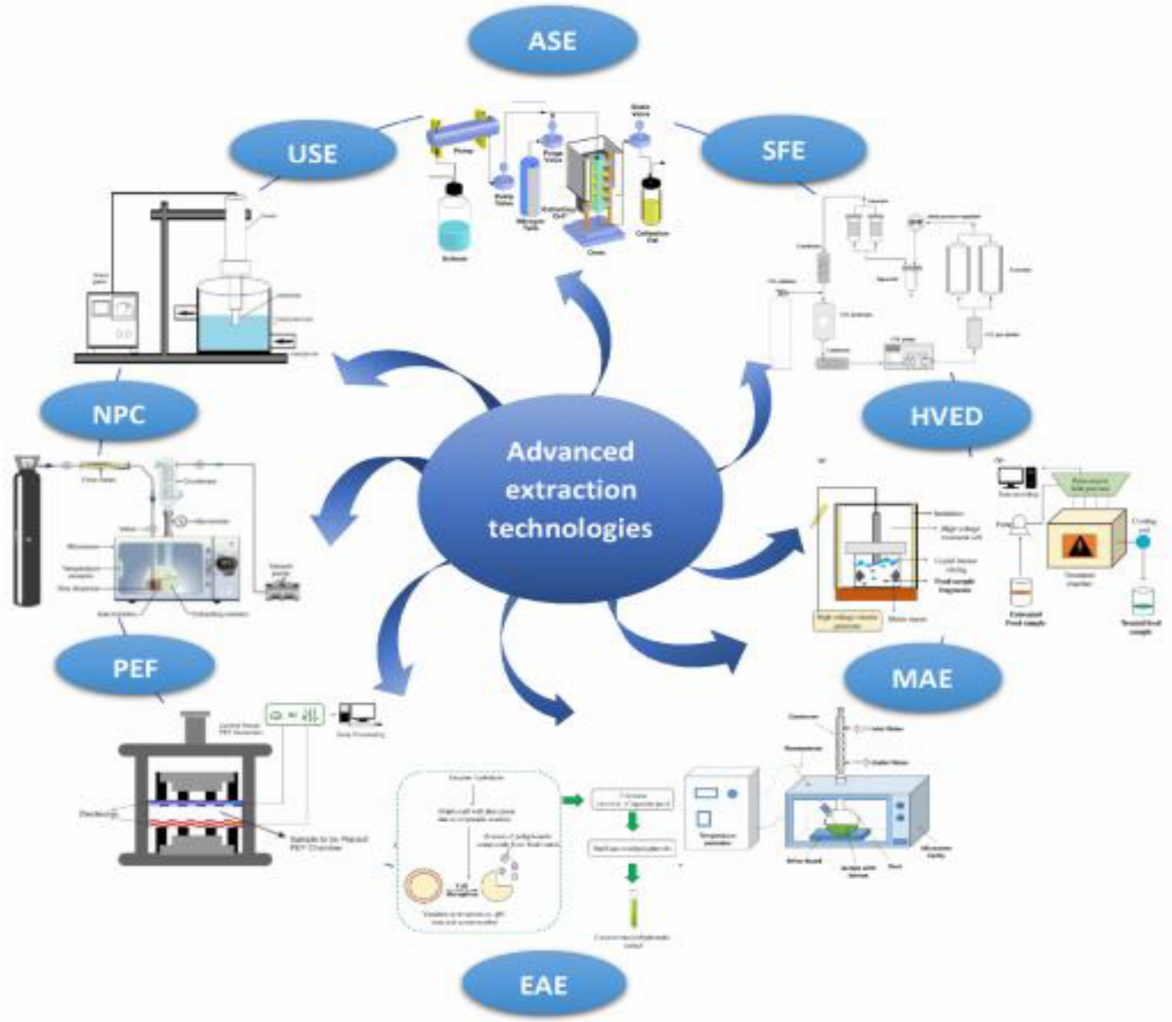
Advanced extraction technologies used for the extraction of bioactive compounds from Artemisia.

As a separation process, plant extraction allows to extract desired soluble metabolites from herb materials. Due to the presence of active components in the herb matrixes, suitable extraction techniques are necessary to extract the active components (Chuo et al., 2022). Conventional methods (like Soxhlet and hydrodistillation extraction as the most widely utilized techniques) due to their drawbacks like degradation of thermolabile components, hydrolysis of EOs, along with long processing time need to be substituted with advanced extraction technologies (Ahmad et al., 2018). There are various new extraction techniques (Figure 1) such as enzyme‐assisted extraction, microwave‐assisted extraction, high‐voltage electrical discharge extraction, ultrasound‐assisted extraction, accelerated solvent extraction, pulsed electric field extraction, supercritical fluid extraction, and negative pressure cavitation which will be evaluated. Among these emerging techniques are those shown below. Table 1 shows the advantages and disadvantages of the application of emerging technologies for the extraction of BACs.

**TABLE 1:** Advantages and disadvantages of the application of emerging technologies for the extraction of bioactive compounds (BACs).

Extraction techniques	Advantages	Disadvantages	Extraction condition	References
UAE	Increase kinetics of extraction and the yield of extractive substance. Time efficient. Not producing toxic components. Reducing solvent and energy consumption	Poor extraction for volatile or nonpolar components. Degradation and oxidation reactions. Nonselective. To avoid excessive temperature, proper power selection is needed. Affects chemical structure of the BACs	0.5–1 h 200–2450 W 25 kHz Organic solvent	Chuo et al. (2022); Zia et al. (2022)
MAE	Time efficient. Increase yield of extraction. Low solvent consumption. Suitable for thermolabile ingredients	Poor extraction for volatile or nonpolar components. Degradation and oxidation reactions. Nonselective. To avoid excessive temperature, proper power selection is needed. Affects chemical structure of the BACs	300 MHz to‐ 300 GHz. Solvents: methanol, water and ethanol	Chuyen et al. (2018)
EAE	Time efficient with low solvent and energy consumption. Higher yield of extraction. Enhancing the extraction of BACs. Improved the antioxidant properties and extraction of soluble phenolics. Eco‐friendly technique	Interactions of food ingredients with enzymes. The stability of enzymes. High price of enzymes. It is hard to scale up to industrial scale	Various enzymes can be utilized, mostly pectinases cellulases, and hemicellulase	Gligor et al. (2019); Wan et al. (2017)
ASE	Increase extraction kinetics. Time efficient. High efficiency by faster penetration of solvent without light and oxygen.	Lower yields of oils extraction utilize of organic solvents. Caramelization and Maillard reactions	Up to 200°C with 3000 psi	Vilas‐Franquesa et al. (2022)
HVDE	Higher extraction yields of high‐added value components. Improve the extraction of phenolic compounds. Lower diffusion time, diffusion temperature, and solvent content	HVED may produce undesirable contaminants, such as free reactive radicals. Metal migration. Producing small particles that are hard to be separated	40–60 kV/cm 2–5 μs	Li et al. (2019)
SFE	Enhance safety and selectivity. Nontoxic organic solvents. Prevent sample oxidization. Time efficient. Sustainable extraction. Higher yield of extraction. Free of inorganic salts and heavy metals. Extracts are very pure	Use high pressure. Need high power. Need very complex and expensive equipment. Polar substances cannot be extracted	45 min‐6 40–80°C 25–40 MPa Cosolvent: Ethanol	Roselló‐Soto et al. (2019); Zia et al. (2022)
PEFE	Increase extraction yield of BACs. Enhance the antioxidant activity. Use low temperature and minimize the degradation of heat‐sensitive components	High cost of the equipment. Need to be thermally controlled to avoid further degradation of the thermos‐sensitive compounds. No inactivation spores. High conductivity values reduce the specificity and effectiveness of the electroporation	10–4 –10 − 2 s 500–1000 V/cm	Martínez et al. (2020); Zia et al. (2022)
NPC	Efficient. Eco‐friendly. Time efficient. Easy large‐scale operation. Avoiding decomposition of thermos‐sensitive components	In large‐scale application, there is concern regarding the efficiency of the technique and the degradation of extract	−0.04 (MPa) pressure; 35 (°C) 35 min Ethanol concentration (v/v): 65%	Kou et al. (2021); Panda and Manickam (2019); Tian et al. (2015)

Abbreviations: ASE, accelerated solvent extraction; EAE, enzyme‐assisted extraction; HVED, High‐voltage electrical discharge extraction; MAE, microwave‐assisted extraction; NPC, Negative pressure cavitation; PEFE, Pulsed electric field extraction; SFE, Supercritical fluid extraction; UAE, ultrasound‐assisted extraction.

#### 2.2.1 Ultrasound‐assisted extraction (UAE)

Ultrasound technique (wave frequency >20 kHz) is a useful, simple, efficient, and economic technology, which does not require expensive instruments (Zia et al., 2022). Ultrasound waves lead to the production of small bubbles during the rarefaction cycles, which grow until the implosion. This implosion generates high temperature and pressure, resulting in cell wall damage and releasing cell contents (Al Khawli et al., 2019; Zia et al., 2022). The efficiency of ultrasound‐assisted extraction is mostly related to temperature, extraction time, plant materials, L/S, and solvent, along with intensity, amplitude, power, and frequency (Chuo et al., 2022). According to results obtained by Patil and Akamanchi (2017) on camptothecin extraction from Nothapodytes nimmoniana, ultrasound‐assisted extraction technique showed higher efficacy (up to 78%) compared to conventional stirring extraction technique (46.5%) (Patil & Akamanchi, 2017). Karabegović et al. (2011) studied the Soxhlet and ultrasonic extraction techniques. They indicated that ultrasound positively affected the kinetics of extraction and the yield of extractive substance, and in the case of both *Artemisia* species (*Artemisia campestris* and *Artemisia vulgaris*) the lowest amounts of flavonoids and total phenolic components are found in the Soxhlet extraction technique which might be related to the longer processing time as well as higher extraction temperature (Karabegović et al., 2011). Total flavonoid and phenolic contents of *A. vulgaris* extracted by SE and ultrasound were 123.4 and 135.0 (total phenolic content/mg·g^−1^), respectively.

#### 2.2.2 Microwave‐assisted extraction (MAE)

Microwave (with frequency between 300 MHz and 300 GHz) is nonionizing electromagnetic radiation (Chuyen et al., 2018). The underlying principle of microwave extraction lies in the energy transfer by an electric field through two coinciding mechanisms. Firstly, to generate heat, energy interacts with the polar molecules in matrix (i.e., water) (Zia et al., 2022). Secondly, during microwave process, ionic conduction moves ions within the solution generating a resistance among ions, which leads to friction and thus heat is produced (Zia et al., 2019). Microwave by the internal pressure and also sudden temperature rise damage the plant cell walls, which help to release the bioactive constituents (Shams et al., 2015). Several factors might influence its efficiency, such as extraction time, dielectric constant, microwave power, solubility, and solvent attributes. According to results obtained by Cardoso‐Ugarte et al. (2014), the yield of extraction through MAE technique was double compared to conventional method (temperature of 80°C) (Cardoso‐Ugarte et al., 2014).

#### 2.2.3 Enzyme‐assisted extraction (EAE)

Unlike high electric field pulses, high hydrostatic pressure compressed, carbon dioxide, and EAE can readily be evaluated on the laboratory scale (Puri et al., 2012). Some compounds are retained by hydrogen or hydrophobic or in the herb matrix and, therefore, are not achievable with common extraction methods (Gligor et al., 2019). To disrupt the structural integrity of the plant cell wall and as a result increase the extraction of bioactives, various enzymes like hemicellulose, pectinases, and cellulases are often required. These enzymes increase cell wall permeability by hydrolyzing cell wall compounds, resulting in higher yields of bioactives (Puri et al., 2012). Elsayed et al. (2022) indicated that the extract obtained by EAE technique (using protease, cellulase, and protease/cellulose (1:1)) had higher ABTS and DPPH free radical inhibition activity as well as higher total phenolic content compared to ethanol extract and BHT (Elsayed et al., 2022). According to Wang et al. (2017), β‐glucosidase or cellulase‐assisted extraction improved the antioxidant activity by 126.5% and the extraction of soluble phenolics by 103.2% (Wang et al., 2017).

#### 2.2.4 Accelerated solvent extraction (ASE)

ASE, with high efficacy in extracting target components from plants, is a relatively new method which uses solvent mixtures or low‐boiling solvents under high temperature and pressure (Hossain et al., 2011). In order to enhance the efficiency of extraction, it uses organic solvents at elevated pressure and temperature (Shams et al., 2015). The high pressure not only allows the extraction cells to be filled faster and helps to force liquid into the solid matrix but also helps to raise the extraction temperature and maintains the solvent in a liquid state (Sirichokworrakit et al., 2022). An increased temperature leads to increased extraction kinetics by augmenting the diffusivity of the solvent (Mandal et al., 2015). According to Sirichokworrakit et al. (2022), the ASE technique is more efficient compared to the UAE and CSE techniques for extracting total phenolic content and total anthocyanin content. Vilas‐Franquesa et al. (2022) compared the ASE technique with conventional method extraction and reported that ASE displayed lower yields of oils. Nevertheless, the oils from ASE revealed greater concentration of monounsaturated fatty acids, polyunsaturated fatty acids, and α‐tocopherol, probably deriving from the greater extraction time in conventional method (Vilas‐Franquesa et al., 2022).

#### 2.2.5 High‐voltage electrical discharge extraction (HVED)

HVED, which is known as corona discharge, is a cold plasma‐based emerging technology. During the HVED process, a gas flows through the needle (electrode), ionizes, and forms a cold plasma. During this process, the permeability of the cell membrane increases, which potentially leads to the formation of membrane pores and higher release of intracellular compounds (Sarkis et al., 2015). Generally, this technique is divided into three categories including; circulating, continuous, as well as batch system with exactly the same mechanisms (the destruction of the cell walls and mass transfer improvement caused by various secondary phenomena, resulting in higher extraction yields of high‐added value components) (Li et al., 2019). The authors indicated that HVED can significantly enhance the extract's antioxidant properties (TEAC and DPPH value) (Boussetta et al., 2011). According to Brianceau et al. (2016), HVED made a remarkable improvement in the phenolic component extraction compared with conventional hydroalcoholic extraction. In the optimized operation conditions, HVED allowed the recovery of 23.6 ± 4.1 mg RES/100 g RM of stilbenes, 56.0 ± 4.5 mg QUE/100 g RM, 794.9 ± 74.1 mg/100 g RM, and 6.6 ± 0.2 mg GAE/100 g RM of stilbenes, flavonols, flavan‐3‐ols, and polyphenols, respectively (Brianceau et al., 2016).

#### 2.2.6 Supercritical fluid extraction (SFE)

The SFE, which uses supercritical fluid as a solvent, is a new and efficient system for the extraction of targeted components from the plants (Zia et al., 2022). Supercritical fluids can easily diffuse through a solid matrix and enhance the extraction rates due to their high diffusivity, low viscosity, as well as enhanced transport properties (de Andrade Lima et al., 2019). In comparison with other extraction methods, SFE enhances selectivity and safety (due to nontoxic organic solvents), prevents sample oxidization, and has less extraction time, resulting in a sustainable extraction (Roselló‐Soto et al., 2019). The solvent utilized in SFE is mostly CO_2_, which is sometimes modified by combination with methanol, ethanol, etc. (Zia et al., 2022). Supercritical CO_2_ is the most commonly utilized solvent in this method, because it is cheap, easily obtainable, nonflammable, nontoxic, achievable critical points (7.39 MPa, 31.1°C), and easy to separate after processing (Chuo et al., 2022). Supercritical fluid can easily transfer active compounds to bulk solvent (due to easy permeation into plant materials) (Chuo et al., 2022). Santos‐Zea et al. (2019), in optimal conditions (60°C, 30 MPa, 10% common solvent (70% ethanol)), investigated *Agave salmiana* and the result of their research led to the optimal extraction of saponin content: 10.95 mg protodioscin/g pl., and antioxidant activity: 17.61 mmol TE/g pl. protodioscin (Santos‐Zea et al., 2019). According to results obtained by Wenqiang et al. (2006) on the extraction of *A. argyi* EOs, the yield of extraction in SFE technique was higher than hydrodistillation technique (0.26%, and 0.20%, respectively). The authors also reported that the presence of coextracted cuticular waxes is the main disadvantage of the oil obtained by SFE (Wenqiang et al., 2006).

#### 2.2.7 Pulsed electric field extraction (PEFE)

During the last decades, PEFE has gained more attention in food waste/by‐products to extract beneficial compounds through drying, pressing, osmosis, and diffusion (Bansal et al., 2015). The main interest of this nonthermal technology, which uses a high‐voltage electric field for a very short time, is cell disruption. This cell disruption can potentially lead to higher membrane permeability, and higher mass transfer of inner liquid and cell compounds from the intracellular vacuoles (Zia et al., 2022). The effectiveness of this method is particularly related to some factors, like pulse intensity, especially energy input, process temperature, strength of field, and sample attributes (Gómez et al., 2019). PEF‐assisted extraction enhanced the antioxidant activity of treated samples as well as improved the release of anthocyanins as compared to untreated samples. Authors studied the PEF as a green technology for the extraction of bioactive components. They indicated that PEF technology in bioactive components extraction from by‐products could be an efficient substitute for conventional techniques which need long extraction times, large amounts of organic solvents, and dried products (Redondo et al., 2018).

#### 2.2.8 Negative pressure cavitation (NPC)

NPC extraction is known as an efficient and eco‐friendly method. In this technology, negative pressure leads to cavitation (created by a vacuum pump) and is continuously introduced into the liquid–solid system. The turbulence, collision, and mass transfer between the extracting solvent and plant matrix are enhanced when air is continuously added into the system via the valet and facilitates the migration of target components out of the matrix. Consequently, secondary metabolites can be rapidly and adequately released into the extraction solvent (Tian et al., 2015). Compared with traditional extraction techniques, NPCE has shown many superiorities, such as higher efficiency, shorter time, easy large‐scale operation, and avoiding decomposition of thermosensitive compounds (Kou et al., 2021). Mu et al. (2012) reported that the extraction yield of vinblastine, vincristine, catharanthine, and vindoline from roseus leaves was 0.126, 0.018, 0.2843, and 0.5783 mg/g DW, respectively, which were higher than the yields obtained by HR and maceration extraction techniques, and equivalent to those obtained from ultrasound extraction method (Mu et al., 2012).

## 3 RESULTS AND DISCUSSION

### 3.1 Plant extracts and EO properties

#### 3.1.1 Antioxidant activity

Quality attributes (texture, flavor, color, organoleptic attributes, nutritional properties, etc.) and shelf life of foods are highly conditioned by the oxidation reactions, particularly those related to lipid oxidation. Furthermore, the use of plant extracts and EOs as preservatives in food products is a growing trend. Different synthetic and natural antioxidants are used to prolong the shelf life of food products, protecting them from lipid oxidation during distribution and sale (Table 2). Various natural and chemical antioxidants are utilized to preserve foods from oxidation reactions and increase their shelf life. Recently, the discussion and concern about whether it is appropriate to utilize synthetic preservatives (such as tertiary butyl hydroquinone, propyl gallate, butylated hydroxyl anisole, and butylated hydroxyl toluene) in foods has enhanced. In this way, actually, it tends to utilize components from natural resources, especially plants. Plants, due to the presence of phenolic constituents such as tannins, phenolic diterpenes, phenolic acids, flavonoids, and phenols, have high antioxidant attributes which act as radical scavengers.

**TABLE 2:** *Artemisia* plants as natural preservatives used in food products.

*Artemisia*	Concentration	Product	Outcomes	References
*A. drAcunculus* EOs (ADEO)	0.25% ADEO	Beef burgers	Reduced the *Staphylococcus aureus* counts by a 2 log CFU/g after 24 h	Chaleshtori et al. (2014)
*A. absinthium* EOs (AEO)	0.15% AEO	–	97% reduction in the optical density of E. coli O157:H7	Rafiq et al. (2016)
*A. fragrans* EOs (AFEO)	1500 ppm AFEO	Chicken fillets	Results indicated 2.27 Log CFU/g for treated samples with AFEO compared to 4.48 Log CFU/g for negative control	
*A. dracunculus* EOs (ADEO)	Chitosan and sodium alginate coating + ADEO	Rainbow trout fillets	On day 1, LAB count was 1.16 log10 CFU/g for CH + ADEO, 1.33 log10 CFU/g for CH coating; On day 12, LAB count was 5.00 log10 CFU/g in control compared to 4.53 log10 CFU/g in CH+ ADEO groups	Raeisi et al. (2020)
*A. dracunculus* EOs (ADEO)	CH (6%) and ADEO (60%)	Yogurt	Primer bacteria and yeast count of yogurt containing CH (6%) and ADEO (60%) were 4.83 and 3.66; in yogurt containing CH (6%) was 5 and 4.16; and in control yogurt were 10.16 and 14 Log CFU/gr, respectively.	Zedan et al. (2021)
*A. dracunculus* EOs (ADEO)	Alginate (AL) and chitosan (CH) coatings containing 0.2% ADEO	Rainbow trout fillets	TBARS values of treated samples with AL + ADEO and CH + ADEO (0.90 and 0.81 mg of malonaldehyde equivalents/kg of tissue, respectively) were significantly lower than treated samples with CH, and control (1.07 and 1.29 mg of malonaldehyde equivalents/kg of tissue)	Raeisi et al. (2020)
*A. fragrans* EOs (AFEO)	2% Calcium Alginate +1500 ppm AFEOs	Chicken meat	CA + AFEOs coating reduced significantly the pH, TVB‐N, and TBARS values. This treatment also displayed a reduction of 3.3 Log CFU/g in molds and yeasts and 2.97 Log CFU/g in coliforms in comparison with control.	
*A. annua* EOs (AAEO)	AAEO and nano zinc oxide	Pork meat	AAEO could effectively inhibit lipid oxidation and microorganism proliferation. AAEO could effectively improve the flexibility, and barrier properties of the films	Zheng et al. (2022)

Ayoughi et al. (2011) assessed the antioxidant properties of *A. dracunculus* L. EOs in soybean oil compared to BHT and BHA. The authors indicated that the antioxidant activity of the BHT and BHA (in 0.2 and 0.01 mg/mL, respectively) with obtained EOs (0.8 mg/mL) had no significant difference (Ayoughi et al., 2011). According to results obtained by Chebbac et al. (2021), *A. negrei* L. EOs displayed a potent DPPH free radical scavenging activity when compared to BHT (IC50 = 0.0164 ± 0.0011 mg/mL and 0.0082 ± 0.002 mg/ mL, respectively) (Chebbac et al., 2021). Chebbac et al. (2022) reported that *A. aragonensis* L. EOs inhibited the DPPH free radicals with an IC50 value of 0.034 ± 0.004 mg/mL, whereas other chemical antioxidants like quercetin, ascorbic acid, and butylated hydroxyl toluene displayed IC50 values of 0.0342 ± 0.002, 0.0124 ± 0.001, and 0.0203 ± 0.005 mg/mL, respectively (Chebbac et al., 2022). Similarly, Devare et al. (2013) reported the high antioxidant attributes of *A. pallens* root extract (DPPH IC50 = 3.013 μg/mL) (Devare et al., 2013). According to results obtained by Moalla et al. (2021), who developed active food packaging films based on chitosan + *A. campestris* (essential oil, aqueous extract, and hydroalcoholic extract), the presence of *A. campestris* significantly enhanced the antioxidant and UV–Vis barrier attributes of the films (Moalla et al., 2021). Lu et al. (2023) also evaluated the antioxidant properties of *A. japonica Thumb* (AJT) extract. The IC50 values of AJT for ABTS+ and DPPH and the OD50 values of AJT for FRAP were determined as 172.37 ± 25.72, 172.37 ± 25.72, and 95.88 ± 5.79 lg/mL, respectively (Lu et al., 2023). Zheng et al. (2022) assessed the effects of chitosan–coix seed starch films containing *A. annua* EOs (AAEO) and nano zinc oxide in pork meat. They reported that AAEO could effectively inhibit lipid oxidation and microorganism proliferation. Results also showed that AAEO could effectively improve the flexibility, and barrier properties of the films (Zheng et al., 2022). Houicher et al. (2013) also reported high antioxidant properties of *A. campestris* extract in vacuum‐packed sardine fillets (Houicher et al., 2013). Raeisi et al. (2020) evaluated the antioxidant attributes of alginate (AL) and chitosan (CH) coatings containing 0.2% *A. dracunculus* EOs (ADEO) in rainbow trout fillets. The results revealed that at the end of storage, the TBARS values of treated samples with AL + ADEO and CH + ADEO (0.90 and 0.81 mg of malonaldehyde equivalents/kg of tissue, respectively) were significantly lower than those treated samples with CH, and control (1.07 and 1.29 mg of malonaldehyde equivalents/kg of tissue); this is because of the antioxidant properties of the extract. TBARS values on day 1 for CH + ADEO were 0.17, AL and AL + ADEO were 0.20, for CH and control was 0.19 mg of malonaldehyde equivalents/kg of tissue, respectively. Artemisia revealed good antimicrobial and antioxidant effects. The authors have indicated that *A. sieberi* possess a great antioxidant potential (Boroomand et al., 2018).  

#### 3.1.2 Antimicrobial properties

Most of the foods are susceptible to spoilage and need to be protected from microbiological growth throughout the process, keeping period and dispensation. There are many techniques that have been utilized to control the microorganisms in foods. The plants’ EOs and extracts are popular to possess antibacterial and fungicidal properties. Hence, they are an efficient and green substitute to synthetic additives, which could decline the risk of food‐borne disease. Nowadays, the EOs and extracts of various *Artemisia* species have become popular, which has encouraged many researchers to evaluate their bioactive components. Components such as para cymene, thymol, carvacrol, and 1, 8 cineole, as the most important compounds of the plants’ EOs (particularly different species of *Artemisia*), have high antimicrobial activities (Massiha et al., 2013). Sharafati Chaleshtori et al. (2014) evaluated the antimicrobial properties of *A. drAcunculus* EOs (ADEO) in beef burgers. They reported that 0.25% ADEO in beef burgers after 24 h reduced the *Staphylococcus aureus* counts by a 2 log CFU/g at 4°C. They also reported that high concentrations of ADEO exhibited more antibacterial activity against *S. aureus*, but their practical consumption is limited due to a negative smell–taste effect in foods (Chaleshtori et al., 2014). Rafiq et al. (2016) also evaluated the antimicrobial activities of *A. absinthium* EOs (AEO) against *E. coli* O157:H7 and *S. typhimurium*. The authors indicated that the presence of 0.15% AEO resulted in a 97% reduction in the optical density of *E. coli* O157:H7, whereas 0.1% AEO were needed to reach a similar reduction in *S. typhimurium*. The AEO at a concentration of 0.2% displayed a 66% reduction in *E. coli* O157:H7 (indicated by 3.04 ± 0.14 log CFU/mL). They reported that at concentrations higher than 0.2%, the bacterial population declined to under detectable levels (Rafiq et al., 2016). Extracts (*Artemisia* L. species) prepared with 90% and 70% ethanol inhibited the growth of G‐negative bacteria (*Pseudomonas aeruginosa*, *Citrobacter freundii*, *Klebsiella ozaenae*, and *Escherichia coli*) and gram‐positive bacteria (*Propionibacterium acnes*, *Enterococcus faecalis*, *streptococci*, and *staphylococci*). Growth inhibition zones ranged from 3.79 to 9.47 mm and 3.79 to 7.33 mm for G‐negative and G‐positive bacteria, respectively, depending on the type of extract and the test culture (Hrytsyk et al., 2021). Raeisi et al. (2020) evaluated the antimicrobial attributes of chitosan (CH) and sodium alginate (SA) coating containing *A. dracunculus* EOs (ADEO) in rainbow trout fillets. The authors reported that on day 1, the lactic acid bacteria count was 1.16 log10 CFU/g for CH + ADEO compared with 1.33 log10 CFU/g for CH coating, indicating good antimicrobial attributes of ADEO. On day 12, LAB values ranged from 5.00 log10 CFU/g in control to 4.53 log10 CFU/g in CH + AD groups (Raeisi et al., 2020). *A. campestris* L. displayed high antimicrobial properties against histidine decarboxylating bacteria (HFB) in fish fillets. High antimicrobial properties of *A. dracunculus* EOs (ADEO) in yogurt were also reported by Zedan et al. (2021). The results showed that primer bacteria and yeast count of yogurt containing CH (6%) and ADEO (60%) were 4.83 and 3.66; in yogurt containing CH (6%) was 5 and 4.16; and in control yogurt were 10.16 and 14 Log CFU/gr, respectively (Zedan et al., 2021). *A. absinthium* extracts showed high antimicrobial activities against *E. coli* O157:H7, *S. typhimurium*, and *L. monocytogenes* in raw beef (Cruz‐Galvez et al., 2013). Artemisia can be used as a novel natural agent for food preservation.

#### 3.1.3 Health and functional properties

Over 260 *Artemisia* species have been evaluated for their secondary metabolites, and essential oils. Results indicated that a total of 23 species of *Artemisia*, with potential antiviral activity (which might be related to the presence of biologically active components), were reported for the treatment of viral diseases (Taleghani et al., 2020). The health benefits of different *Artemisia* species are presented in Table 3. Among the reported *Artemisia* species, *A. annua* extracts (aqueous, ethanol, and methanol extracts) due to presence of artemisinin, as an antiviral and antimalarial compound, have proven to be widely utilized against various viruses such as HBV, HIV‐1, SARS‐CoV‐2, BVDV, Influenza virus type A (IV‐A), and HHV (HSV‐1) (Hussain, 2022). Scopoletin and isoscopoletin, which are found widely in different species of *Artemisia* extracts, were proven to efficiently postpone cancer progression (Song et al., 2019). The results indicated that the oral administration of *A. herba alba* extract (300 mg/kg) led to significant reductions in tumor weight (similar to those reported in cisplatin and artesunate‐treated groups) (Mohamed et al., 2020). The authors indicated that borneol not only can accelerate brain distribution and intestinal absorption but also causes the effective ingredients of Sanqi and Danshen to play a quicker therapeutic role and enhance the therapeutic effect (Lv et al., 2018). Anti‐inflammation and immunomodulation activity of *A. argyi* have been reported to act as general immune system stimulators to increase the function of natural killer cells B and T lymphocytes as well as enhancing host defense responses through regulating the secretion of antibodies and cytokines (Song et al., 2019). *A. argyi* have a wide range of pharmacological effects such as immunomodulatory, antiosteoporotic, anticoagulant, anti‐inflammatory, antitumor, and antioxidant activities (Ge et al., 2016; Lv et al., 2018; Yun et al., 2016; Zhang et al., 2018). For example, according to results obtained by Zhang et al. (2018) extracted polysaccharides from *A. argyi* increased the levels of cytokines (IL‐1β, IL‐6, and TNF‐α) and immunoglobulins (IgM and IgG), suggesting the immunomodulation activity of *A. argyi* polysaccharides. *Artemisia* species are pharmaceutically used by people for their anticancer (Gupta et al., 2022), anti‐inflammatory (Miranda et al., 2022), and antithrombotic (Subramani & Sathiyarajeswaran, 2022) properties. *Artemisia* species with strong anti‐inflammatory and antioxidant properties can enhance vascular health (Shen et al., 2022). Amyrins are triterpenes (found in *Artemisia*) that have hypoglycemic and anti‐inflammatory activity along with a gastro‐protective effect (Carvalho et al., 2017).

**TABLE 3:** Health benefits of *Artemisia* species.

*Artemisia* species	Health benefits	Reference
*A. annua*	Utilized against various viruses such as HBV, HIV‐1, SARS‐CoV‐2, BVDV, Influenza virus type A (IV‐A), and HHV (HSV‐1). It has anticholesterolemic, antimicrobial, anti‐inflammatory, anticonvulsant, antiplasmodial, antitumor, and antihyperlipidemic properties	Hussain (2022); Wang et al. (2020)
*A. vulgaris*	It is utilized as infusions to which stomachic, cytostatic, antibacterial, anthelmintic, antitumor, and antipyretic actions have been attributed and postpone cancer progression	Blagojević et al. (2006)
*A. herba alba*	Reducing tumor weight; treat stomach problems such as abdominal pain and diarrhea and healing exterior wounds; has antifungal, antispasmodic, and antibacterial activities, counter the threat of COVID‐19; DNA destruction and reducing cell viability in cancer cells and has shown a potential cytotoxic activity against different cancer	Brahmi et al. (2022); Hasan et al. (2022); Kadri et al. (2022)
*A. pallens*	Treatment of blood pressure, depression, cold, cough, and measles and diabetes, with high anti‐inflammatory activity	Pavithra et al. (2018)
*A. argyi*	Anti‐inflammation and immunomodulation activity; increase the function of natural killer cells B and T lymphocytes; secretion of antibodies and cytokines, immunomodulatory, antiosteoporotic, anticoagulant, anti‐inflammatory, antitumor, and antioxidant activities, treatment of menstrual‐related symptoms, hemostasis, diarrhea, and eczema	Liu et al. (2021); Lv et al. (2018); Shin et al. (2017); Song et al. (2019); Zhang et al. (2018)
*A absinthium*	Treating stomach ache, worms, fevers, and sepsis, and act as a diuretic, stimulant, carminative, digestive, antidepressant, antiseptic, and choleretic impacts	Koul and Taak (2018)
*A. afra*	Treat different sicknesses such as dyspepsia, malaria, coughs, diabetes, cold, headaches, and disorders of bladder and kidney	Patil et al. (2011)
*A. japonica*	Treat tuberculosis, hypertension, malaria, headache, and fever, beneficial for liver disorders such as jaundice and viral hepatitis	Lu et al. (2023)

## 4 PREVIOUS CONSIDERATIONS FOR USING *ARTEMISIA* SPECIES IN FOODS

When an extraction process comes to scaling and the extracted compounds are utilized in foods, the laws have great significance. For the implementation of these extracted compounds in food industry, the legal dose which can be utilized is listed among the most vital issues (Pateiro et al., 2018). In Europe, with regard to the process condition of foods (thermally treated or not) and their fat content, Regulation 1129/2011 sets the limits for each of these compounds as well as allowed components (Commission, 2011). Different countries have different legal considerations; however, their implementation of foods as safer ingredients implies its consideration as GRAS (Food and Drug Administration, 2003). FDA has a list of many GRAS EOs that can be utilized in foods. Furthermore, the application of *Artemisia* plant in foods should not alter the technological attributes of the foods (the most important properties that need to be taken into account are the organoleptic properties and consumer acceptance). There are many researches that suggest the possibility of utilizing natural EOs and extracts instead of chemical ingredients. However, to achieve the same results as chemical preservatives, higher concentrations of natural preservatives are needed, but it may alter the organoleptic properties of products. To overcome this issue, the combination of natural components could be an efficient technique to enhance their antimicrobial and/or antioxidant properties and increase the stability of food products because of their possible synergistic effects, decreasing the negative physiological and organoleptic side effects. On the other hand, an antagonistic effect might also happen (low activity when components are utilized in combination with together compared with individual impacts) (Tajik et al., 2014).

## 5 IMPLEMENTATION OF *ARTEMISIA* SPECIES EXTRACTION PRODUCTS IN FOOD INDUSTRY

Nowadays, production of functional and healthier food products that can help promote consumer's well‐being, due to their demands, needs to be taken into account (Putnik et al., 2017). The application of natural ingredients in the production of functional foods can be a useful technique. In this way, *Artemisia* species in a concentrated form (as EOs and/or extracts) can be used in food products. Due to the declared utilization of natural additives, which are able to decrease microbial growth and oxidation reactions (leading to clean‐label food products), these technological processes could be well accepted (Granato et al., 2017). The application forms of these ingredients in products (nanoparticles, directly, active packaging, etc.) is a key factor for the effectiveness and stability of the new formulation. Depending on the food products, the EOs can be utilized as nanoparticles, directly or in combination with packaging. Spray drying, which is listed among the most common techniques for encapsulation, not only can mask unacceptable odors and control the release of active compounds in the food products, but also can protect encapsulated compounds (EOs) against processing conditions (storage time, temperature, pH, exposure to light and oxygen, etc.) (Pateiro et al., 2018). As a result, compounds protected by layers of a coating agent, resulting in better quality attributes. Active food packaging, which provides a new system for food preservation in the food industry, is an innovative technology. Active packaging is prepared by incorporating active agents, particularly natural EOs and extracts, into packaging materials and works by releasing active agents (releasing these compounds in a controlled manner potentially enhances its efficacy, resulting in higher stability) into the surrounding environment. These compounds can be applied to the substrate of active food packaging either in encapsulated form (electro spun fibers, microbeads, spray‐dried microcapsules, etc.) or free form (in the form of w/w, w/o, or o/w emulsions) (Vilela et al., 2018).

## 6 FUTURE DIRECTIONS AND CONCLUSIONS

Increasing consumer demands for healthy food products led to a progressed interest in food industries to substitute chemical additives with natural antioxidants and antimicrobial compounds to maintain quality and freshness. The use of *Artemisia* EOs and extracts as active packaging and nanoparticles in foods can be an efficient method to decline the application of synthetic antioxidants and preservatives. The efficacy of herb derivatives has been proved in various research; however, there are few natural antioxidants and antimicrobials commercially available. This phenomenon is mainly related to the various extraction techniques throughout the production processes and storage as well as various phenolic components in plants’ EOs and extracts. Hence, to maintain volatile components among preparation and achieve complete bioactive components extraction, the optimizing and modeling of extraction could be an efficient method. The use of antioxidant and antimicrobial active packaging, which has been proposed as a good substitute to traditional food packaging, could increase the stability, shelf‐life, and of quality foods. Hence, the application of natural antioxidant and antimicrobials not only provides high protective activity on lipid oxidation and microbiological spoilage but also satisfies consumer demands for the substitution of synthetic additives. The application of novel extraction techniques would allow us to obtain high‐value bioactive components along with protecting both the consumers and environment. This would give value to the processed products by the industry from *Artemisia* plant materials to bioactive compound extraction, combination in foods aits sale. Prior to use in foods, more studies are needed to evaluate clinical trials to better evaluate Artemisia plants effects on health. These studies need to be completed with researches that assess their bioavailability and bioaccessibility in the consumer's body.

## AUTHOR CONTRIBUTIONS

**Negin Ahmadi: Writing – original draft (lead). Nazanin Mosleh:** Funding acquisition (equal). **Mahta Yeganeh:** Resources (equal). **Nadia Ahmadi:** Writing – original draft (equal). Sara Malakouti: Resources (equal). Saleh Shahsavari: Resources (equal). Reza Shahraki: Funding acquisition (equal) Somaye Katebi: Funding acquisition (equal). Mina Agapoor: Funding acquisition (equal). **Sonia Sadeghi:** Writing – review and editing (equal). Karim Bagheri: Writing – review and editing (equal).

## CONFLICT OF INTEREST STATEMENT

The authors declare no conflict of interest relevant to this article.

## Data Availability

The data are available upon request from the authors.

## References

[fsn34303-bib-0001] Ahmad, A. , Hamidah Mohd‐Setapar, S. , Karakucuk, A. , Azim, M. M. , Asyikin, Z. , Aziz, A. , Hamidah, S. , Setapar, M. , Lokhat, D. , Rafatullah, M. , Ganash, M. , Kamal, M. A. , & Ashraf, G. M. (2018). Essential oils: Extraction techniques, pharmaceutical and therapeutic potential‐a review. Current Drug Metabolism, 19. 1100–1110. 10.2174/1389200219666180723144850 30039757

[fsn34303-bib-0002] Al Khawli, F. , Pateiro, M. , Domínguez, R. , Lorenzo, J. M. , Gullón, P. , Kousoulaki, K. , Ferrer, E. , Berrada, H. , & Barba, F. J. (2019). Innovative green technologies of intensification for valorization of seafood and their by‐products. Marine Drugs, 17(12), 689. 31817754 10.3390/md17120689 PMC6950251

[fsn34303-bib-0007] Al‐Zubairi, A. S. , Abdul, A. B. , Abdelwahab, S. I. , Peng, C. Y. , Mohan, S. , & Elhassan, M. M. (2011). *Eleucine indica* possesses antioxidant, antibacterial and cytotoxic properties. Evidence‐based Complementary and Alternative Medicine, 2011, 965370. 19617201 10.1093/ecam/nep091 PMC3137868

[fsn34303-bib-0008] Anwar, F. , Ahmad, N. , Alkharfy, K. M. , & Gilani, A.‐u.‐H. (2016). Mugwort (*Artemisia vulgaris*) oils. In V. R. Preedy (Ed.), Essential oils in food preservation, flavor and safety (pp. 573–579). Elsevier.

[fsn34303-bib-0009] Asdadi, A. , Hamdouch, A. , Gharby, S. , & Hassani, L. M. I. (2020). Chemical characterization of essential oil of *Artemisia herba‐alba* asso and his possible potential against covid‐19. Journal of Analytical Sciences and Applied Biotechnology, 2(2), 2.

[fsn34303-bib-0010] Ayoughi, F. , Marzegar, M. , Sahari, M. A. , & Naghdibadi, H. (2011). Chemical compositions of essential oils of *Artemisia dracunculus* L. and endemic *Matricaria chamomilla* L. and an evaluation of their antioxidative effects. Journal of Agricultural Science and Technology, 13(1), 79–88.

[fsn34303-bib-0011] Babayan, A. M. , Petrosyan, M. T. , & Sahakyan, N. Z. (2022). The chemical composition and antioxidant properties of some species of Artemisia genus, represented in Armenian flora. Proceedings of the YSU B: Chemical and Biological Sciences, 56(2), 161–168.

[fsn34303-bib-0012] Baldino, L. , Reverchon, E. , & Della Porta, G. (2017). An optimized process for SC‐CO_2_ extraction of antimalarial compounds from *Artemisia annua* L. The Journal of Supercritical Fluids, 128, 89–93.

[fsn34303-bib-0013] Bansal, V. , Sharma, A. , Ghanshyam, C. , Singla, M. L. , & Kim, K.‐H. (2015). Influence of pulsed electric field and heat treatment on *Emblica officinalis* juice inoculated with *Zygosaccharomyces bailii* . Food and Bioproducts Processing, 95, 146–154.

[fsn34303-bib-0014] Belhattab, R. , Amor, L. , Barroso, J. G. , Pedro, L. G. , & Figueiredo, A. C. (2014). Essential oil from *Artemisia herba‐alba* Asso grown wild in Algeria: Variability assessment and comparison with an updated literature survey. Arabian Journal of Chemistry, 7(2), 243–251.

[fsn34303-bib-0015] Blagojević, P. , Radulović, N. , Palić, R. , & Stojanović, G. (2006). Chemical composition of the essential oils of Serbian wild‐growing *Artemisia absinthium* and *Artemisia vulgaris* . Journal of Agricultural and Food Chemistry, 54(13), 4780–4789. 16787028 10.1021/jf060123o

[fsn34303-bib-0016] Bora, K. S. , & Sharma, A. (2011). The genus Artemisia: A comprehensive review. Pharmaceutical Biology, 49(1), 101–109. 20681755 10.3109/13880209.2010.497815

[fsn34303-bib-0017] Boroomand, N. , Sadat‐Hosseini, M. , Moghbeli, M. , & Farajpour, M. (2018). Phytochemical components, total phenol and mineral contents and antioxidant activity of six major medicinal plants from Rayen, Iran. Natural Product Research, 32(5), 564–567. 28403651 10.1080/14786419.2017.1315579

[fsn34303-bib-0018] Boussetta, N. , Vorobiev, E. , Deloison, V. , Pochez, F. , Falcimaigne‐Cordin, A. , & Lanoisellé, J.‐L. (2011). Valorisation of grape pomace by the extraction of phenolic antioxidants: Application of high voltage electrical discharges. Food Chemistry, 128(2), 364–370. 25212143 10.1016/j.foodchem.2011.03.035

[fsn34303-bib-0019] Brahmi, F. , Iblhoulen, Y. , Issaadi, H. , Elsebai, M. F. , Madani, K. , & Boulekbache‐Makhlouf, L. (2022). Ethnobotanical survey of medicinal plants of Bejaia localities from Algeria to prevent and treat coronavirus (COVID‐19) infection shortened title: Phytomedicine to manage COVID‐19 pandemic. Advances in Traditional Medicine, 23, 1–13.

[fsn34303-bib-0020] Brianceau, S. , Turk, M. , Vitrac, X. , & Vorobiev, E. (2016). High voltage electric discharges assisted extraction of phenolic compounds from grape stems: Effect of processing parameters on flavan‐3‐ols, flavonols and stilbenes recovery. Innovative Food Science & Emerging Technologies, 35, 67–74.

[fsn34303-bib-0021] Cardoso‐Ugarte, G. A. , Sosa‐Morales, M. E. , Ballard, T. , Liceaga, A. , & San Martín‐González, M. F. (2014). Microwave‐assisted extraction of betalains from red beet (*Beta vulgaris*). LWT‐ Food Science and Technology, 59(1), 276–282.

[fsn34303-bib-0022] Carvalho, K. M. M. B. , de Melo, T. S. , de Melo, K. M. , Quinderé, A. L. G. , de Oliveira, F. T. B. , Viana, A. F. S. C. , Nunes, P. I. G. , da Silva Quetz, J. , de Araújo Viana, D. , & Havt, A. (2017). Amyrins from *Protium heptaphyllum* reduce high‐fat diet‐induced obesity in mice via modulation of enzymatic, hormonal and inflammatory responses. Planta Medica, 83(3/4), 285–291. 27525508 10.1055/s-0042-114222

[fsn34303-bib-0023] Chaleshtori, R. S. , Rokni, N. , Rafieian‐Kopaei, M. , Drees, F. , Sharafati‐Chaleshtori, A. , & Salehi, E. (2014). Use of tarragon (*Artemisia dracunculus*) essential oil as a natural preservative in beef burger. Italian Journal of Food Science, 26, 427–432.

[fsn34303-bib-0024] Chebbac, K. , Ghneim, H. K. , El Moussaoui, A. , Bourhia, M. , El Barnossi, A. , Benziane Ouaritini, Z. , Salamatullah, A. M. , Alzahrani, A. , Aboul‐Soud, M. A. M. , & Giesy, J. P. (2022). Antioxidant and antimicrobial activities of chemically‐characterized essential oil from *Artemisia aragonensis* Lam. against drug‐resistant microbes. Molecules, 27(3), 1136. 35164402 10.3390/molecules27031136 PMC8840534

[fsn34303-bib-0025] Chebbac, K. , Moussaoui, A. E. L. , Bourhia, M. , Salamatullah, A. M. , Alzahrani, A. , & Guemmouh, R. (2021). Chemical analysis and antioxidant and antimicrobial activity of essential oils from *Artemisia negrei* L. against drug‐resistant microbes. Evidence‐based Complementary and Alternative Medicine, 2021, 5902851. 34539801 10.1155/2021/5902851 PMC8443344

[fsn34303-bib-0026] Chuo, S. C. , Nasir, H. M. , Mohd‐Setapar, S. H. , Mohamed, S. F. , Ahmad, A. , Wani, W. A. , Muddassir, M. , & Alarifi, A. (2022). A glimpse into the extraction methods of active compounds from plants. Critical Reviews in Analytical Chemistry, 52(4), 667–696. 32954795 10.1080/10408347.2020.1820851

[fsn34303-bib-0027] Chuyen, H. V. , Nguyen, M. H. , Roach, P. D. , Golding, J. B. , & Parks, S. E. (2018). Microwave‐assisted extraction and ultrasound‐assisted extraction for recovering carotenoids from Gac peel and their effects on antioxidant capacity of the extracts. Food Science & Nutrition, 6(1), 189–196. 29387378 10.1002/fsn3.546 PMC5778220

[fsn34303-bib-0028] Commission, E. U . (2011). Commission regulation (EU) No 1129/2011 of 11 November 2011 amending annex II to regulation (EC) No 1333/2008 of the European Parliament and of the council by establishing a union list of food additives. Official Journal of the European Union L, 295(4), 11–12.

[fsn34303-bib-0029] Cruz‐Galvez, A. M. , Gómez‐Aldapa, C. A. , Villagómez‐Ibarra, J. R. , Chavarría‐Hernández, N. , Rodríguez‐Baños, J. , Rangel‐Vargas, E. , & Castro‐Rosas, J. (2013). Antibacterial effect against foodborne bacteria of plants used in traditional medicine in central Mexico: Studies in vitro and in raw beef. Food Control, 32(1), 289–295.

[fsn34303-bib-0030] de Andrade Lima, M. , Kestekoglou, I. , Charalampopoulos, D. , & Chatzifragkou, A. (2019). Supercritical fluid extraction of carotenoids from vegetable waste matrices. Molecules, 24(3), 466. 30696092 10.3390/molecules24030466 PMC6384789

[fsn34303-bib-0031] Devare, S. M. , Patil, J. A. , Gaikwad, S. A. , Torne, R. C. , Deshpande, N. R. , & Salvekar, J. P. (2013). Antioxidant potential of *Artemisia pallens* roots. International Journal of PharmTech Research, 5, 1360–1363.

[fsn34303-bib-0032] Dongare, S. (2022). *Artemisia pallens*: An Indian plant with multifarious pharmacological potentials. International Journal of Medical & Pharmaceutical Sciences, 12(2), 1.

[fsn34303-bib-0033] du Toit, A. , & van der Kooy, F. (2019). *Artemisia afra*, a controversial herbal remedy or a treasure trove of new drugs? Journal of Ethnopharmacology, 244, 112127. 31376515 10.1016/j.jep.2019.112127

[fsn34303-bib-0034] Durgadevi, M. , Narayanapur, V. B. , Vishwanath, Y. C. , Hegde, L. , Bhuvaneshwari, G. , & Gandolkar, K. (2022). Davana a potential under exploited aromatic crop of south India: A review. Pharmaceutical Innovation, 11(2), 22–27.

[fsn34303-bib-0035] Ekiert, H. , Pajor, J. , Klin, P. , Rzepiela, A. , Ślesak, H. , & Szopa, A. (2020). Significance of *Artemisia vulgaris* L. (Common Mugwort) in the history of medicine and its possible contemporary applications substantiated by phytochemical and pharmacological studies. Molecules, 25(19), 4415. 32992959 10.3390/molecules25194415 PMC7583039

[fsn34303-bib-0036] Elsayed, N. , Marrez, D. A. , Ali, M. A. , El‐Maksoud, A. A. A. , Cheng, W. , & Abedelmaksoud, T. G. (2022). Phenolic profiling and in‐vitro bioactivities of corn (*Zea mays* L.) tassel extracts by combining enzyme‐assisted extraction. Food, 11(14), 2145. 10.3390/foods11142145 PMC9320485 35885388

[fsn34303-bib-0037] Feng, X. , Cao, S. , Qiu, F. , & Zhang, B. (2020). Traditional application and modern pharmacological research of *Artemisia annua* L. Pharmacology & Therapeutics, 216, 107650. 32758647 10.1016/j.pharmthera.2020.107650

[fsn34303-bib-0038] Food and Drug Administration . (2003). Essential oils, oleoresins (solvent free), and natural extractives including distillates. Code of Federal Regulations.

[fsn34303-bib-0040] Ge, Y. , Wang, Z. , Xiong, Y. , Huang, X. , Mei, Z. , & Hong, Z. (2016). Anti‐inflammatory and blood stasis activities of essential oil extracted from *Artemisia argyi* leaf in animals. Journal of Natural Medicines, 70, 531–538. 26894818 10.1007/s11418-016-0972-6

[fsn34303-bib-0042] Gligor, O. , Mocan, A. , Moldovan, C. , Locatelli, M. , Crișan, G. , & Ferreira, I. C. F. R. (2019). Enzyme‐assisted extractions of polyphenols–a comprehensive review. Trends in Food Science & Technology, 88, 302–315.

[fsn34303-bib-0043] Gómez, B. , Munekata, P. E. S. , Gavahian, M. , Barba, F. J. , Martí‐Quijal, F. J. , Bolumar, T. , Campagnol, P. C. B. , Tomasevic, I. , & Lorenzo, J. M. (2019). Application of pulsed electric fields in meat and fish processing industries: An overview. Food Research International, 123, 95–105. 31285034 10.1016/j.foodres.2019.04.047

[fsn34303-bib-0044] Goud, B. J. , Dwarakanath, V. , & Swamy, B. K. (2015). A review on history, controversy, traditional use, ethnobotany, phytochemistry and pharmacology of *Artemisia absinthium* Linn. International Journal of Advanced Research in Engineering and Applied Sciences, 4(5), 77–107.

[fsn34303-bib-0045] Granato, D. , Nunes, D. S. , & Barba, F. J. (2017). An integrated strategy between food chemistry, biology, nutrition, pharmacology, and statistics in the development of functional foods: A proposal. Trends in Food Science & Technology, 62, 13–22.

[fsn34303-bib-0046] Gupta, J. , Ahuja, A. , & Gupta, R. (2022). Green approaches for cancers management: An effective tool for health care. Anti‐Cancer Agents in Medicinal Chemistry (Formerly Current Medicinal Chemistry‐Anti‐Cancer Agents), 22(1), 101–114. 10.2174/1871520621666210119091826 33463475

[fsn34303-bib-0047] Haile, A. B. , & Jiru, T. M. (2022). Antibacterial effects of *Artemisia afra* leaf crude extract against some selected multi‐antibiotic resistant clinical pathogens. Ethiopian Journal of Health Sciences, 32(3), 651. 35813676 10.4314/ejhs.v32i3.22 PMC9214731

[fsn34303-bib-0048] Han, B. , Xin, Z. , Ma, S. , Liu, W. , Zhang, B. , Ran, L. , Yi, L. , & Ren, D. (2017). Comprehensive characterization and identification of antioxidants in folium *Artemisiae argyi* using high‐resolution tandem mass spectrometry. Journal of Chromatography B, 1063, 84–92. 10.1016/j.jchromb.2017.08.021 28850890

[fsn34303-bib-0049] Hasan, A. , Biswas, P. , Bondhon, T. A. , Jannat, K. , Paul, T. K. , Paul, A. K. , Jahan, R. , Nissapatorn, V. , Mahboob, T. , & Wilairatana, P. (2022). Can *Artemisia herba‐alba* be useful for managing COVID‐19 and comorbidities? Molecules, 27(2), 492. 35056809 10.3390/molecules27020492 PMC8779608

[fsn34303-bib-0050] Hossain, M. B. , Barry‐Ryan, C. , Martin‐Diana, A. B. , & Brunton, N. P. (2011). Optimisation of accelerated solvent extraction of antioxidant compounds from rosemary (*Rosmarinus officinalis* L.), marjoram (*Origanum majorana* L.) and oregano (*Origanum vulgare* L.) using response surface methodology. Food Chemistry, 126(1), 339–346.

[fsn34303-bib-0051] Houicher, A. , Kuley, E. , Bendeddouche, B. , & Özogul, F. (2013). Effect of *Mentha spicata* L. and *Artemisia campestris* extracts on the shelf life and quality of vacuum‐packed refrigerated sardine (*Sardina pilchardus*) fillets. Journal of Food Protection, 76(10), 1719–1725. 24112571 10.4315/0362-028X.JFP-13-118

[fsn34303-bib-0052] Hrytsyk, R. A. , Kutsyk, R. V. , Yurchyshyn, O. I. , Struk, О. А. , Kireev, I. V. , & Grytsyk, A. R. (2021). The investigation of antimicrobial and antifungal activity of some *Artemisia* L. species. Pharmacia, 68(1), 93–100.

[fsn34303-bib-0054] Hussain, A. (2022). A phylogenetic perspective of antiviral species of the genus Artemisia (Asteraceae‐anthemideae): A proposal of anti SARS‐CoV‐2 (COVID‐19) candidate taxa. Journal of Herbal Medicine, 36, 100601. 36188629 10.1016/j.hermed.2022.100601 PMC9514968

[fsn34303-bib-0055] Jiang, Z. , Guo, X. , Zhang, K. , Sekaran, G. , Cao, B. , Zhao, Q. , Zhang, S. , Kirby, G. M. , & Zhang, X. (2019). The essential oils and eucalyptol from *Artemisia vulgaris* L. prevent acetaminophen‐induced liver injury by activating Nrf2–Keap1 and enhancing APAP clearance through non‐toxic metabolic pathway. Frontiers in Pharmacology, 10, 782. 31404264 10.3389/fphar.2019.00782 PMC6669816

[fsn34303-bib-0056] Judžentienė, A. , & Buzelytė, J. (2006). Chemical composition of essential oils of *Artemisia vulgaris* L. (mugwort) from North Lithuania. Chemija, 17(1), 12–15.

[fsn34303-bib-0057] Kadri, M. , Yahia, A. , Goubi, S. , Mekhedmi, N. E. , Selmane, M. , & Chemsa, A. E. (2022). Chromatography analysis, in vitro antioxidant and antibacterial activities of essential oil of *Artemisia herba‐alba* Asso of Boussaâda, Algeria. Biodiversitas Journal of Biological Diversity, 23(9), 4424–4431.

[fsn34303-bib-0058] Karabegović, I. , Nikolova, M. , Veličković, D. , Stojičević, S. , Veljković, V. , & Lazić, M. (2011). Comparison of antioxidant and antimicrobial activities of methanolic extracts of the Artemisia sp. recovered by different extraction techniques. Chinese Journal of Chemical Engineering, 19(3), 504–511.

[fsn34303-bib-0059] Kazeminia, M. , Mehrabi, A. , & Mahmoudi, R. (2022). Chemical composition, biological activities, and nutritional application of Asteraceae family herbs: A systematic review. Trends in Phytochemical Research, 6(3):187–213.

[fsn34303-bib-0060] Koshinski, R. (2018). Effect of *Artemisia annua* L. extract on growth performance, biochemical blood parameters and meat quality of rainbow trout (*Oncorhynchus mykiss* W.), cultivated in recirculating system. Agricultural Science & Technology (1313‐8820), 10(3), 266.

[fsn34303-bib-0061] Kou, P. , Wang, S. X. , Pan, H. Y. , Wan, N. , Wang, X. Q. , Liu, Z. G. , Zhao, C. J. , Jiang, S. G. , & Fu, Y. J. (2021). Preparative separation of specific triterpenoids from *Inonotus obliquus* based on negative‐pressure cavitation extraction and high‐speed counter‐current chromatography. Journal of the Taiwan Institute of Chemical Engineers, 125, 69–77. 10.1016/j.jtice.2021.06.024

[fsn34303-bib-0062] Koul, B. , & Taak, P. (2018). The Artemisia genus: A review on traditional uses, phytochemical constituents, pharmacological properties and germplasm conservation. Journal of Glycomics & Lipidomics, 7(1), 1–7. 10.4172/2153-0637.1000142

[fsn34303-bib-0063] Kumar, A. P. , & Kumud, U. (2010). Pharmacognostic and phytochemical investigation of aerial parts of *Artemisia pallens* wall ex. Dc. Pharmacognosy Journal, 2(9), 285–288.

[fsn34303-bib-0064] Li, Z. , Fan, Y. , & Xi, J. (2019). Recent advances in high voltage electric discharge extraction of bioactive ingredients from plant materials. Food Chemistry, 277, 246–260. 30502141 10.1016/j.foodchem.2018.10.119

[fsn34303-bib-0065] Liu, Y. , He, Y. , Wang, F. , Xu, R. , Yang, M. , Ci, Z. , Wu, Z. , Zhang, D. , & Lin, J. (2021). From longevity grass to contemporary soft gold: Explore the chemical constituents, pharmacology, and toxicology of *Artemisia argyi* H. Lév. & vaniot essential oil. Journal of Ethnopharmacology, 279, 114404. 34246739 10.1016/j.jep.2021.114404

[fsn34303-bib-0066] Lu, Y. , Li, S. , Lai, Q. , Wang, L. , Zhou, W. , Hua, C. , Ning, D. , Zhang, C. , Li, M. , & Jiang, F. (2023). Chemical constituents, antioxidant and hepatoprotective properties of ethanol extract from *Artemisia japonica* thumb. Leaves. Arabian Journal of Chemistry, 16(3), 104526.

[fsn34303-bib-0067] Lv, J.‐L. , Li, Z.‐Z. , & Zhang, L.‐B. (2018). Two new flavonoids from *Artemisia argyi* with their anticoagulation activities. Natural Product Research, 32(6), 632–639. 28539062 10.1080/14786419.2017.1332603

[fsn34303-bib-0068] Mandal, S. C. , Mandal, V. , & Das, A. K. (2015). Essentials of botanical extraction: Principles and applications. Academic Press.

[fsn34303-bib-0069] Martínez, J. M. , Delso, C. , Álvarez, I. , & Raso, J. (2020). Pulsed electric field‐assisted extraction of valuable compounds from microorganisms. Comprehensive Reviews in Food Science and Food Safety, 19(2), 530–552. 33325176 10.1111/1541-4337.12512

[fsn34303-bib-0070] Massiha, A. , Khoshkholgh‐Pahlaviani, M. M. , Issazadeh, K. , Bidarigh, S. , & Zarrabi, S. (2013). Antibacterial activity of essential oils and plant extracts of Artemisia (*Artemisia annua* L.) in vitro. Zahedan Journal of Research in Medical Sciences, 15(6), e92933.

[fsn34303-bib-0071] Mehani, M. , Segni, L. , Terzi, V. , Morcia, C. , Ghizzoni, R. , Goudgil, B. , & Benchikh, S. (2018). Antifungal activity of *Artemisia herba‐alba* on various fusarium. Phytothérapie, 16(2), 87–90.

[fsn34303-bib-0072] Miranda, R. d. S. , de Jesus, B. d. S. M. , da Silva Luiz, S. R. , Viana, C. B. , Adao Malafaia, C. R. , Figueiredo, F. d. S. , Carvalho, T. d. S. C. , Silva, M. L. , Londero, V. S. , & da Costa‐Silva, T. A. (2022). Antiinflammatory activity of natural triterpenes—An overview from 2006 to 2021. Phytotherapy Research, 36(4), 1459–1506. 35229374 10.1002/ptr.7359

[fsn34303-bib-0073] Mirghaed, A. T. , Paknejad, H. , & Mirzargar, S. S. (2020). Hepatoprotective effects of dietary Artemisia (*Artemisia annua*) leaf extract on common carp (*Cyprinus carpio*) exposed to ambient ammonia. Aquaculture, 527, 735443.

[fsn34303-bib-0074] Moalla, S. , Ammar, I. , Fauconnier, M.‐L. , Danthine, S. , Blecker, C. , Besbes, S. , & Attia, H. (2021). Development and characterization of chitosan films carrying *Artemisia campestris* antioxidants for potential use as active food packaging materials. International Journal of Biological Macromolecules, 183, 254–266. 33892038 10.1016/j.ijbiomac.2021.04.113

[fsn34303-bib-0075] Mohamed, A. E.‐H. H. , El‐Sayed, M. , Hegazy, M. E. , Helaly, S. E. , Esmail, A. M. , & Mohamed, N. S. (2010). Chemical constituents and biological activities of *Artemisia herba‐alba* . Records of Natural Products, 4(1), 1–25.

[fsn34303-bib-0076] Mohamed, H. R. H. , Amer, M. , & Faky, A. S. A. E. (2020). Growth retardation and apoptotic death of tumor cells by *Artemisia herba‐alba* oral administration in Ehrlich solid carcinoma bearing mice. Revista Brasileira de Farmacognosia, 29, 763–772.

[fsn34303-bib-0077] Mohammed, S. , Dekabo, A. , & Hailu, T. (2022). Phytochemical analysis and anti‐microbial activities of Artemisia spp. and rapid isolation methods of artemisinin. AMB Express, 12(1), 17. 35150378 10.1186/s13568-022-01346-5 PMC8840944

[fsn34303-bib-0078] Moufid, A. , & Eddouks, M. (2012). *Artemisia herba alba*: A popular plant with potential medicinal properties. Pakistan Journal of Biological Sciences: PJBS, 15(24), 1152–1159. 23755405 10.3923/pjbs.2012.1152.1159

[fsn34303-bib-0079] Mu, F. , Yang, L. , Wang, W. , Luo, M. , Fu, Y. , Guo, X. , & Zu, Y. (2012). Negative‐pressure cavitation extraction of four main vinca alkaloids from *Catharanthus roseus* leaves. Molecules, 17(8), 8742–8752. 22832876 10.3390/molecules17088742 PMC6268964

[fsn34303-bib-0080] Panda, D. , & Manickam, S. (2019). Cavitation technology—The future of greener extraction method: A review on the extraction of natural products and process intensification mechanism and perspectives. Applied Sciences, 9(4), 766.

[fsn34303-bib-0081] Pandey, B. P. , Thapa, R. , & Upreti, A. (2017). Chemical composition, antioxidant and antibacterial activities of essential oil and methanol extract of *Artemisia vulgaris* and *Gaultheria fragrantissima* collected from Nepal. Asian Pacific Journal of Tropical Medicine, 10(10), 952–959. 29111190 10.1016/j.apjtm.2017.09.005

[fsn34303-bib-0082] Pateiro, M. , Barba, F. J. , Domínguez, R. , Sant'Ana, A. S. , Khaneghah, A. M. , Gavahian, M. , Gómez, B. , & Lorenzo, J. M. (2018). Essential oils as natural additives to prevent oxidation reactions in meat and meat products: A review. Food Research International, 113, 156–166. 30195508 10.1016/j.foodres.2018.07.014

[fsn34303-bib-0083] Patel, D. K. (2022). Biological potential and therapeutic benefit of Chrysosplenetin: An applications of polymethoxylated flavonoid in medicine from natural sources. Pharmacological Research – Modern Chinese Medicine, 4, 100155.

[fsn34303-bib-0084] Patil, D. M. , & Akamanchi, K. G. (2017). Microwave assisted process intensification and kinetic modelling: Extraction of camptothecin from *Nothapodytes nimmoniana* plant. Industrial Crops and Products, 98, 60–67.

[fsn34303-bib-0085] Patil, G. V. , Dass, S. K. , & Chandra, R. (2011). *Artemisia afra* and modern diseases. J Pharmacogenom Pharmacoproteomics, 2(105), 645–2153.

[fsn34303-bib-0086] Pavithra, K. S. , Annadurai, J. , & Ragunathan, R. (2018). Phytochemical, antioxidant and a study of bioactive compounds from *Artemisia pallens* . Journal of Pharmacognosy and Phytochemistry, 7(4), 664–675.

[fsn34303-bib-0087] Puri, M. , Sharma, D. , Barrow, C. J. , & Tiwary, A. K. (2012). Optimisation of novel method for the extraction of steviosides from *Stevia rebaudiana* leaves. Food Chemistry, 132(3), 1113–1120. 29243590 10.1016/j.foodchem.2011.11.063

[fsn34303-bib-0088] Putnik, P. , Barba, F. J. , Lorenzo, J. M. , Gabrić, D. , Shpigelman, A. , Cravotto, G. , & Bursać Kovačević, D. (2017). An integrated approach to mandarin processing: Food safety and nutritional quality, consumer preference, and nutrient bioaccessibility. Comprehensive Reviews in Food Science and Food Safety, 16(6), 1345–1358. 33371593 10.1111/1541-4337.12310

[fsn34303-bib-0089] Raeisi, M. , Hashemi, M. , Aminzare, M. , Ghorbani Bidkorpeh, F. , Ebrahimi, M. , Jannat, B. , Tepe, B. , & Noori, S. M. A. (2020). Effects of sodium alginate and chitosan coating combined with three different essential oils on microbial and chemical attributes of rainbow trout fillets. Journal of Aquatic Food Product Technology, 29(3), 253–263.

[fsn34303-bib-0090] Rafiq, R. , Hayek, S. A. , Anyanwu, U. , Hardy, B. I. , Giddings, V. L. , Ibrahim, S. A. , Tahergorabi, R. , & Kang, H. W. (2016). Antibacterial and antioxidant activities of essential oils from *Artemisia herba‐alba* Asso., *Pelargonium capitatum* × radens and *Laurus nobilis* L. Food, 5(2), 28. 10.3390/foods5020028 PMC5302350 28231123

[fsn34303-bib-0091] Redondo, D. , Venturini, M. E. , Luengo, E. , Raso, J. , & Arias, E. (2018). Pulsed electric fields as a green technology for the extraction of bioactive compounds from thinned peach by‐products. Innovative Food Science & Emerging Technologies, 45, 335–343.

[fsn34303-bib-0092] Roselló‐Soto, E. , Barba, F. J. , Lorenzo, J. M. , Dominguez, R. , Pateiro, M. , Mañes, J. , & Moltó, J. C. (2019). Evaluating the impact of supercritical‐CO_2_ pressure on the recovery and quality of oil from “horchata” by‐products: Fatty acid profile, α‐tocopherol, phenolic compounds, and lipid oxidation parameters. Food Research International, 120, 888–894. 31000310 10.1016/j.foodres.2018.11.054

[fsn34303-bib-0093] Santos‐Zea, L. , Gutiérrez‐Uribe, J. A. , & Benedito, J. (2019). Effect of ultrasound intensification on the supercritical fluid extraction of phytochemicals from *Agave salmiana* bagasse. The Journal of Supercritical Fluids, 144, 98–107.

[fsn34303-bib-0094] Sapkota, S. , Kadariya, I. P. , Pandey, M. , Risal, P. , & Basnet, B. B. (2022). Antioxidant activity of essential oil of *Artemisia vulgaris* collected from sub‐tropical region of Bagmati province, Nepal. Journal of Agriculture and Forestry University, 5, 203–207.

[fsn34303-bib-0095] Sarkis, J. R. , Boussetta, N. , Tessaro, I. C. , Marczak, L. D. F. , & Vorobiev, E. (2015). Application of pulsed electric fields and high voltage electrical discharges for oil extraction from sesame seeds. Journal of Food Engineering, 153, 20–27.

[fsn34303-bib-0096] Shahrivari, S. , Alizadeh, S. , Ghassemi‐Golezani, K. , & Aryakia, E. (2022). A comprehensive study on essential oil compositions, antioxidant, anticholinesterase and antityrosinase activities of three Iranian Artemisia species. Scientific Reports, 12(1), 7234. 35508595 10.1038/s41598-022-11375-6 PMC9068787

[fsn34303-bib-0097] Shams, K. A. , Abdel‐Azim, N. S. , Saleh, I. A. , Hegazy, M. F. , El‐Missiry, M. M. , Hammouda, F. M. , Bohouth, E. , & Tahrir, E. (2015). Green technology: Economically and environmentally innovative methods for extraction of medicinal & aromatic plants (MAP) in Egypt. Journal of Chemical and Pharmaceutical Research, 7(5), 1050–1074.

[fsn34303-bib-0098] Shen, N. , Wang, T. , Gan, Q. , Liu, S. , Wang, L. , & Jin, B. (2022). Plant flavonoids: Classification, distribution, biosynthesis, and antioxidant activity. Food Chemistry, 383, 132531. 35413752 10.1016/j.foodchem.2022.132531

[fsn34303-bib-0099] Shin, N.‐R. , Ryu, H.‐W. , Ko, J.‐W. , Park, S.‐H. , Yuk, H.‐J. , Kim, H.‐J. , Kim, J.‐C. , Jeong, S.‐H. , & Shin, I.‐S. (2017). *Artemisia argyi* attenuates airway inflammation in ovalbumin‐induced asthmatic animals. Journal of Ethnopharmacology, 209, 108–115. 28735728 10.1016/j.jep.2017.07.033

[fsn34303-bib-0100] Sirichokworrakit, S. , Rimkeeree, H. , Chantrapornchai, W. , & Sukatta, U. (2022). Comparative study on conventional, accelerated solvent extraction and ultrasonic‐assisted extraction of total phenolic and anthocyanin contents and antioxidant activities from Riceberry bran. Agriculture and Natural Resources, 56(2), 243–254.

[fsn34303-bib-0101] Skiker, M. , Mekhfi, H. , Aziz, M. , Haloui, B. , Lahlou, S. , Legssyer, A. , Bnouham, M. , & Ziyyat, A. (2010). *Artemisia herba‐alba* Asso relaxes the rat aorta through activation of NO/cGMP pathway and KATP channels. Journal of Smooth Muscle Research, 46(3), 165–174. 20647693 10.1540/jsmr.46.165

[fsn34303-bib-0102] Soares, M. P. , Cardoso, I. L. , Ishikawa, M. M. , de Oliveira, A. d. S. S. , Sartoratto, A. , Jonsson, C. M. , de Queiroz, S. C. d. N. , Duarte, M. C. T. , Rantin, F. T. , & Sampaio, F. G. (2020). Effects of *Artemisia annua* alcohol extract on physiological and innate immunity of Nile tilapia (*Oreochromis niloticus*) to improve health status. Fish & Shellfish Immunology, 105, 369–377. 32693158 10.1016/j.fsi.2020.07.035

[fsn34303-bib-0103] Song, X. , Wen, X. , He, J. , Zhao, H. , Li, S. , & Wang, M. (2019). Phytochemical components and biological activities of *Artemisia argyi* . Journal of Functional Foods, 52, 648–662.

[fsn34303-bib-0104] Subramani, B. , & Sathiyarajeswaran, P. (2022). Current update on herbal sources of antithrombotic activity—A comprehensive review. The Egyptian Journal of Internal Medicine, 34(1), 1–12. 10.1186/s43162-021-00090-9 PMC8899788 35283622

[fsn34303-bib-0105] Szopa, A. , Pajor, J. , Klin, P. , Rzepiela, A. , Elansary, H. O. , Al‐Mana, F. A. , Mattar, M. A. , & Ekiert, H. (2020). *Artemisia absinthium* L.—Importance in the history of medicine, the latest advances in phytochemistry and therapeutical, cosmetological and culinary uses. Plants, 9(9), 1063. 32825178 10.3390/plants9091063 PMC7570121

[fsn34303-bib-0106] Tajik, H. , Farhangfar, A. , Moradi, M. , & Razavi Rohani, S. M. (2014). Effectiveness of clove essential oil and grape seed extract combination on microbial and lipid oxidation characteristics of raw buffalo patty during storage at abuse refrigeration temperature. Journal of Food Processing and Preservation, 38(1), 31–38.

[fsn34303-bib-0107] Taleghani, A. , Emami, S. A. , & Tayarani‐Najaran, Z. (2020). Artemisia: A promising plant for the treatment of cancer. Bioorganic & Medicinal Chemistry, 28(1), 115180. 31784199 10.1016/j.bmc.2019.115180

[fsn34303-bib-0108] Tian, H. , Li, W.‐Y. , Xiao, D. , Li, Z.‐M. , & Wang, J.‐W. (2015). Negative‐pressure cavitation extraction of secoisolariciresinol diglycoside from flaxseed cakes. Molecules, 20(6), 11076–11089. 26083040 10.3390/molecules200611076 PMC6272233

[fsn34303-bib-0109] Tilaoui, M. , Ait Mouse, H. , Jaafari, A. , & Zyad, A. (2015). Comparative phytochemical analysis of essential oils from different biological parts of *Artemisia herba alba* and their cytotoxic effect on cancer cells. PLoS One, 10(7), e0131799. 26196123 10.1371/journal.pone.0131799 PMC4510584

[fsn34303-bib-0110] Van Wyk, B.‐E. (2008). A broad review of commercially important southern African medicinal plants. Journal of Ethnopharmacology, 119(3), 342–355. 18577439 10.1016/j.jep.2008.05.029

[fsn34303-bib-0111] Van Wyk, B.‐E. (2011). The potential of south African plants in the development of new medicinal products. South African Journal of Botany, 77(4), 812–829.

[fsn34303-bib-0112] Vilas‐Franquesa, A. , Saldo, J. , & Juan, B. (2022). Sea buckthorn (*Hippophae rhamnoides*) oil extracted with hexane, ethanol, diethyl ether and 2‐MTHF at different temperatures–an individual assessment. Journal of Food Composition and Analysis, 114, 104752.

[fsn34303-bib-0113] Vilela, C. , Kurek, M. , Hayouka, Z. , Röcker, B. , Yildirim, S. , Antunes, M. D. C. , Nilsen‐Nygaard, J. , Pettersen, M. K. , & Freire, C. S. R. (2018). A concise guide to active agents for active food packaging. Trends in Food Science & Technology, 80, 212–222.

[fsn34303-bib-0114] Vo, V. C. (1997). Dictionary of Vietnamese medicinal plants (1249). Medical Publishing House.

[fsn34303-bib-0115] Wan, X. L. , Song, Z. H. , Niu, Y. , Cheng, K. , Zhang, J. F. , Ahmad, H. , Zhang, L. L. , & Wang, T. (2017). Evaluation of enzymatically treated *Artemisia annua* L. on growth performance, meat quality, and oxidative stability of breast and thigh muscles in broilers. Poultry Science, 96(4), 844–850. 10.3382/ps/pew307 27608659

[fsn34303-bib-0116] Wang, D. , Cui, L. , Chang, X. , & Guan, D. (2020). Biosynthesis and characterization of zinc oxide nanoparticles from *Artemisia annua* and investigate their effect on proliferation, osteogenic differentiation and mineralization in human osteoblast‐like MG‐63 cells. Journal of Photochemistry and Photobiology B: Biology, 202, 111652. 31760374 10.1016/j.jphotobiol.2019.111652

[fsn34303-bib-0117] Wang, L. , Wu, Y. , Liu, Y. , & Wu, Z. (2017). Complex enzyme‐assisted extraction releases antioxidative phenolic compositions from guava leaves. Molecules, 22(10), 1648. 28973991 10.3390/molecules22101648 PMC6151667

[fsn34303-bib-0118] Wenqiang, G. , Shufen, L. , Ruixiang, Y. , & Yanfeng, H. (2006). Comparison of composition and antifungal activity of *Artemisia argyi* Levl. et Vant inflorescence essential oil extracted by hydrodistillation and supercritical carbon dioxide. Natural Product Research, 20(11), 992–998. 17032625 10.1080/14786410600921599

[fsn34303-bib-0121] Yamari, A. , Boriky, D. , Bouamrani, M. L. , Blaghen, M. , & Talbi, M. (2004). A new thiophen acetylene from *Artemisia absinthium* . Journal of the Chinese Chemical Society, 51(3), 637–638.

[fsn34303-bib-0122] Yan, L. , Xiong, C. , Xu, P. , Zhu, J. , Yang, Z. , Ren, H. , & Luo, Q. (2019). Structural characterization and in vitro antitumor activity of A polysaccharide from *Artemisia annua* L. (Huang Huahao). Carbohydrate Polymers, 213, 361–369. 30879680 10.1016/j.carbpol.2019.02.081

[fsn34303-bib-0123] Yimam, B. B. , & Desalew, A. (2022). Phytochemical screening, antibacterial effect, and essential oil extract from the leaf of *Artemisia afra* against on selected pathogens. Advances in Microbiology, 12(7), 386–397.

[fsn34303-bib-0124] Yun, C. , Jung, Y. , Chun, W. , Yang, B. , Ryu, J. , Lim, C. , Kim, J.‐H. , Kim, H. , & Cho, S.‐I. (2016). Anti‐inflammatory effects of Artemisia leaf extract in mice with contact dermatitis in vitro and in vivo. Mediators of Inflammation, 2016, 1–8. 10.1155/2016/8027537 PMC5018339 27647952

[fsn34303-bib-0125] Zeb, S. , Ali, A. , Zaman, W. , Zeb, S. , Ali, S. , Ullah, F. , & Shakoor, A. (2019). Pharmacology, taxonomy and phytochemistry of the genus Artemisia specifically from Pakistan: A comprehensive review. Pharmaceutical and Biomedical Research, 4(4), 1–12.

[fsn34303-bib-0126] Zedan, H. , Hosseini, S. M. , & Mohammadi, A. (2021). The effect of tarragon (*Artemisia dracunculus*) essential oil and high molecular weight chitosan on sensory properties and shelf life of yogurt. LWT, 147, 111613.

[fsn34303-bib-0127] Zhang, P. , Shi, B. , Li, T. , Xu, Y. , Jin, X. , Guo, X. , & Yan, S. (2018). Immunomodulatory effect of *Artemisia argyi* polysaccharide on peripheral blood leucocyte of broiler chickens. Journal of Animal Physiology and Animal Nutrition, 102(4), 939–946. 29604137 10.1111/jpn.12895

[fsn34303-bib-0128] Zheng, K. , Zhang, J. , Yang, F. , Wang, W. , Li, W. , & Qin, C. (2022). Properties and biological activity of chitosan‐coix seed starch films incorporated with nano zinc oxide and *Artemisia annua* essential oil for pork preservation. LWT, 164, 113665.

[fsn34303-bib-0129] Zia, S. , Khan, M. R. , Shabbir, M. A. , Aslam Maan, A. , Khan, M. K. I. , Nadeem, M. , Khalil, A. A. , Din, A. , & Aadil, R. M. (2022). An inclusive overview of advanced thermal and nonthermal extraction techniques for bioactive compounds in food and food‐related matrices. Food Reviews International, 38(6), 1166–1196.

[fsn34303-bib-0130] Zia, S. , Khan, M. R. , Zeng, X. , Shabbir, M. A. , & Aadil, R. M. (2019). Combined effect of microwave and ultrasonication treatments on the quality and stability of sugarcane juice during cold storage. International Journal of Food Science & Technology, 54(8), 2563–2569.

